# Long-Time Persisting Superhydrophilicity on Sapphire Surface via Femtosecond Laser Processing with the Varnish of TiO_2_

**DOI:** 10.3390/nano12193403

**Published:** 2022-09-28

**Authors:** Dandan Yan, Zhi Yu, Tingting Zou, Yucai Lin, Wenchi Kong, Jianjun Yang

**Affiliations:** 1 GPL Photonics Laboratory, State Key Laboratory of Applied Optics, Changchun Institute of Optics, Fine Mechanics and Physics (CIOMP), Chinese Academy of Sciences (CAS), Changchun 130033, China; 2 Center of Materials Science and Optoelectronics Engineering, University of Chinese Academy of Sciences, Beijing 100049, China

**Keywords:** femtosecond laser, long-time superhydrophilicity, sapphire, TiO_2_

## Abstract

The acquiring of superhydrophilic surfaces attracts the strong interest in self-cleaning, anti-fogging and anti-icing fields based on the unique features. However, the persistent time of superhydrophilic surfaces is still facing a big challenge because of easily adsorbing hydrophobic groups. Here, we propose a strategy to achieve a superhydrophilicity persisting for an unprecedently long time on sapphire surfaces, by compounding the femtosecond laser-induced hierarchical structures and the subsequent varnish of TiO_2_. The superhydrophilic effect (with a contact angle of CA = 0°) created by our method can be well prolonged to at least 180 days, even for its storage in air without additional illumination of UV lights. Based on comprehensive investigations, we attribute the underlying mechanisms to the coordination of laser-induced metal ions on the material surface via TiO_2_ doping, which not only prevents the adsorption of the nonpolar hydrocarbon groups, but also modulates the photo-response properties of TiO_2_. In addition, further experiments demonstrate the excellent anti-fogging properties of our prepared samples. This investigation provides a new perspective for further enhancing the durability of superhydrophilicity surfaces.

## 1. Introduction

Over decades of years, the superhydrophilicity of material surfaces has attracted great attention in many fields, because of its real significance not only in the fundamental research but also for practical applications such as self-cleaning, anti-fogging, anti-icing, and fluid transport [1,2,3,4,5,6,7,8]. In order to achieve this target, several different methods have been implemented. One is a deposition of molecular films with the pronounced hydrophilic property on the substrate; for example, thiols are commonly used to enhance the surface hydrophilicity of gold, silver, and copper materials [9,10,11,12,13]. The second hydrophilic technique is the hydrophilic treatment by plasma, photons, electrons, ions, and ozone [14,15,16,17,18], which often exhibit disadvantages in both the mechanical strength and the time aging, thus actually impeding their practical applications.

During recent years, the femtosecond laser has proven to be a new powerful tool for high-precision manufacturing, and its unique modifications on both the surface morphology and physicochemical properties demonstrate many potentials in optics, solar-thermal energy technologies, and wettability [19,20,21,22]. Usually, however, the lifetime of the obtained superhydrophilic surfaces by laser processing has been found very limited, i.e., for the direct exposing of the laser-processed materials to air, the available surface superhydrophilicity can be maintained only for a few days or even serval hours [23,24,25]. Such a phenomenon has been attributed by many researchers to the adsorption of hydrophobic groups on the structured surfaces [26,27,28,29]. In order to settle this problem, Christina et al. tried to increase the surface roughness and surface energy of steel surfaces using the femtosecond laser ablation in a nitrogen environment, resulting in the hydrophilic surface with the contact angle of CA = 24 ± 3° for 101 days [30]. On the other hand, by in situ deposition of SiO_2_ on 316 L stainless steel with the nanosecond laser, Rajab et. al reported the superhydrophilic behavior (CA < 2°) lasting for six months, which benefits from the high surface energy of silicon and oxygen contents [31]. More recently, Rajan et al. demonstrated the sustainable superhydrophilic property (CA = 0°) for about 42 days, when the zeolite films were coated on the surface of aluminum alloys treated by the femtosecond laser [32].

As a matter of fact, the wide bandgap semiconductor TiO_2_ is another category of hydrophilic materials [33], and it was found to display the superhydrophilicity upon irradiation with UV light. By considering the wide potential applications in environmental purification and solar energy conversion [34,35], this discovery has further aroused great research interest. However, such wettability conversion often suffers from certain limitations: the essentially required optical absorption from high-energy photons to create electron-hole pairs because of the large bandgap (3.2 eV) for TiO_2_; the short lifetime of superhydrophilicity by the recombination of charge carriers after UV irradiation. For example, the UV-induced superhydrophilic of TiO_2_ films began to fail after storing in darkness for about 10 days, with gradual increasing of the contact angle from CA = 0° to 53° [36].

To suppress the recombination of photogenerated charge carriers, Watanabe et. al. developed a novel TiO_2_/WO_3_ film to improve sensitivity of the superhydrophilic but still with UV illumination, and its superhydrophilic durability was yet unknown [37]. On the other hand, many metal and non-metal ions dopants have been investigated for TiO_2_ to improve the photocatalytic activity [38,39,40,41], while few such works were reported on the change of hydrophilicity. In general, seeking a sustainable superhydrophilic effect, especially under no UV light irradiation, is a substantial challenge in current situations.

Based on the above analysis, we here propose a novel strategy of the manipulation on the sapphire surface by femtosecond laser processing associated with the varnish of TiO_2_, to successfully achieve long-time persisting (at least for 180 days) superhydrophilicity with CA = 0°. Specifically, the micro/nano-structures were firstly prepared on the sapphire surface by femtosecond laser to show the superhydrophilicity with CA = 0°. Then, the TiO_2_ precursor solution was spin-coated on its surface and annealed by high temperature to obtained anatase with Al^3+^ ions doping, which tends to inhibit the subsequent adsorption of hydrophobic groups. Meanwhile, our sample surface could exhibit a high absorption throughout the UV-Vis range. As a result, an extremely long-lasting superhydrophilicity on the sapphire surfaces could be surprisingly observed. Furthermore, our superhydrophilic surface is experimentally shown to have great anti-fogging potential.

## 2. Materials and Methods

### 2.1. Preparation of Materials

Substrate material: A single crystal sapphire wafer (crystal face <0001>) was employed as a sample material, because of its excellent physical, chemical, and optical properties for the wide applications.

Synthesis of TiO_2_ precursor: First, HCl solution (35% concentration, CHRON CHEMICALS, Chengdu, China) was diluted to 2 M with deionized water. Then 35 μL 2 M HCl was mixed with 2.53 mL anhydrous ethanol (CH₃CH₂OH, ≥99.7% concentration, Sinopharm Chemical Reagent, Shanghai, China) in air, which is denoted as solution A. Secondly, 369 μL titanium isopropoxide ((CH_3_CH_3_CHO)_4_Ti, 95% concentration, Shanghai Aladdin Biochemical Technology, Shanghai, China) was added into 2.53 mL anhydrous ethanol in a glove box, which is denoted as solution B. Finally, solutions A and B were mixed and stirred for 12 h at room temperature.

### 2.2. Femtosecond Laser Processing

**Figure 1:**
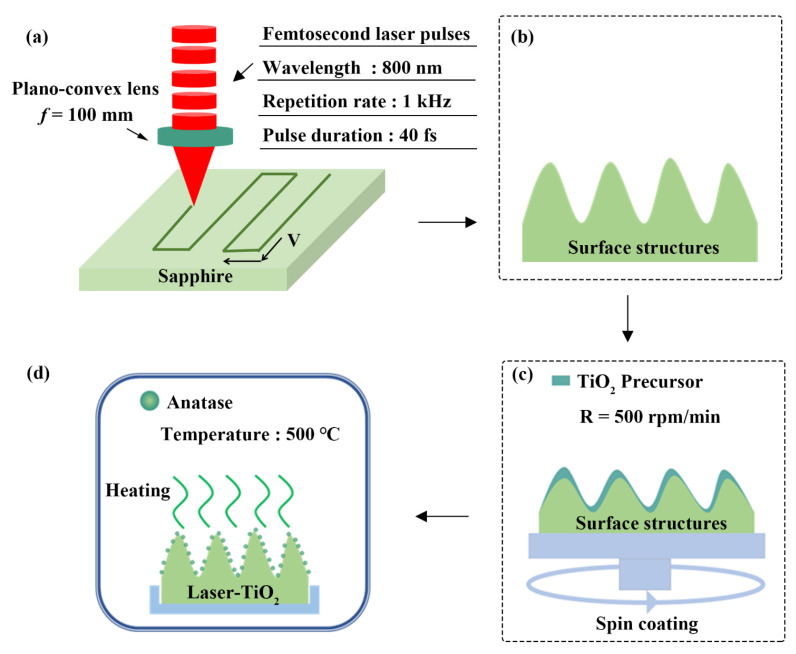
(**a**,**b**) Schematic diagrams of the femtosecond laser processing on the sapphire surface; (**c**,**d**) Preparation procedures for the varnish of TiO_2_ onto the sapphire surface micro/nano-structured by the femtosecond laser.

A schematic diagram of the experimental setup for the femtosecond laser processing is shown in Figure 1a,b, where a commercial chirped pulse amplification system of Ti: Sapphire laser (Spitfire Ace, Spectra Physics, Milpitas, CA, USA) was employed as a light source at a repetition rate of 1 kHz, with a central wavelength of 800 nm and a pulse time duration of 40 fs. Each laser pulse delivered from the laser system has the maximum energy of 7 mJ with the linear polarization. In our practical experiments, the laser-pulse energy reduced to a small value of 0.2 mJ by passing through variable attenuators, and then it was focused onto the sapphire surface by a plano-convex optical lens with a focal length of f = 100 mm. The diameter of the focused laser beam was calculated by Zemax software (ANSYS, Canonsburg, PA, USA) to be 12 ± 0.6 μm, with the corresponding laser energy fluence of F = 155 ± 7.75 J/cm^2^. The spatial interval between the two adjacent lines was of 35 μm and the scanning speed was varied as V = 0.1, 0.3, 0.5 mm/s. The direction of the sample scanning was parallel to the laser polarization. Variable micro/nano-structures can be produced on the sapphire surface with different laser-scanning speeds (see Figure S1 from the Supplementary Materials). All the laser-processing experiments were carried out in the atmosphere environment.

### 2.3. Varnishing TiO_2_ Film on the Sapphire Surface after Laser Processing

The TiO_2_ precursor was spin-coated onto the laser-ablated sapphire surface for 30 s at a rotation speed R = 500 rpm/min, then the sample was annealed at 500 °C for 2 h in a muffle furnace, as shown in Figure 1c,d.

### 2.4. Characterizations

The crystalline structure of the materials was characterized by an X-ray diffractometer (XRD, Bruker D8 Advance, Karlsruhe, Germany), with a measurement range of 2θ = 20–90°. The surface morphology was analyzed by a field emission scanning electron microscope (SEM, Zeiss, Oberkochen, Germany). Elemental distribution was measured by using the Energy Dispersive Spectrometer (EDS, Zeiss, Oberkochen, Germany). The chemical compositions of the materials were analyzed by X-ray photoelectron spectroscopy (XPS, Thermo Fisher Scientific, Waltham, MA, USA), whose source gun type is Al K Alpha with a stepping energy of 0.05 eV. A video surveillance-based contact angle measuring instrument (Powereach, Shanghai, China) was used to characterize the surface wettability. The optical absorption of the samples was measured with a UV-Vis-NIR spectrophotometer (Agilent, Santa Clara, CA, USA).

## 3. Results and Discussion

### 3.1. Superhydrophilicity Obtained on Sapphire Surface by Laser Processing

At first, we prepared a superhydrophilic surface with CA = 0° (see video S1 from the Supplementary Materials) on the sapphire material by the laser ablation processing. However, we found an increase in the contact angle with its storage time in air (Figure S2 from the Supplementary Materials), which means that such superhydrophilic property of CA = 0° is only temporary and cannot be maintained in a long term. According to the previous studies [42,43], the adsorption of nonpolar hydrocarbon groups on the material surface is also responsible for the transition from the superhydrophilic to the superhydrophobic. The observation of this fact in our experiments was shown in Figure S3a–c (from the Supplementary Materials), where the XPS measurement of C 1 s spectra on the material surfaces can be decomposed into four individual Gaussian curves, with the identified binding energy peaks of 284.6 eV, 286.1 eV, 289.1 eV and 291.6 eV. Correspondingly, they belong to the functional groups of C-C/C-H, C-O, COO^−^ and π = π* shake-up, respectively [44,45]. Because the C-C/C-H bonds are characterized by the non-polar properties, their emergence is responsible for the hydrophobic behavior of the material surface. In other words, the accumulative adsorption of non-polar C-C/C-H bonds would like to gradually deteriorate the superhydrophilicity on the material surface. Now a more general question is raised concerning how to effectively prevent the adsorption of such a non-polar hydrophobic group on the material surface, so that the superhydrophilic phenomenon will persist in a long time.

**Figure 2:**
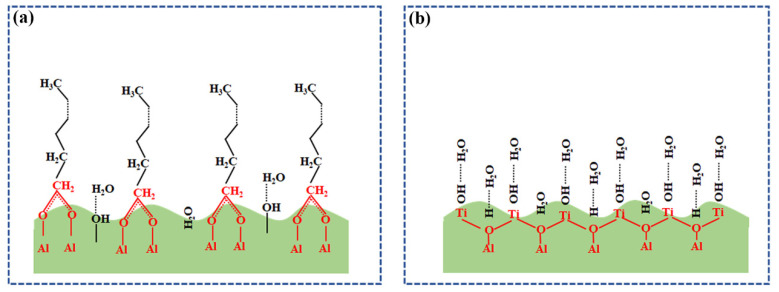
Schematic diagram for depiction of the superhydrophilic evolvement on the sapphire surface under different conditions. (**a**) Chemisorption of non-polar saturated hydrocarbon chains on the laser-treated surface to make the superhydrophilic persistence shorted and conversion into the hydrophobicity; (**b**) Coordination of metal ions on the laser-structured sapphire surface by TiO_2_ varnish to allow the adsorption of hydrophilic group for extending the superhydrophilic durability.

Usually, after the femtosecond laser interaction with the materials, some uncoordinated metal cations can be created on the surface to result in the strong electron acceptability. In our case, there should be a massive Al^3+^ cations generated on the sapphire surface through the hot nonequilibrium plasma formation, which is produced by the femtosecond laser processing. As a result, the carboxylate radical anions COO^−^ can be naturally absorbed with the surface cations Al^3+^, and then develop into a stable chemisorbed carboxylate layer [46], as shown in Figure 2a. Because the saturated hydrocarbon chains extending outward from the carboxylate layer turn to be non-polar, both the wettability and the subsequent adsorption behaviors of the surface will be affected. When the non-polar saturated hydrocarbon chains continue to adsorb the non-polar groups in air with the lapse of time, a dense physically adsorbed hydrophobic layer can be formed on the sample surface. Under such circumstances, the superhydrophilicity is apt to converse into the hydrophobic.

### 3.2. Superhydrophilic Persistence Improved by the Varnish of TiO_2_

Based on the above analyses, we suppose that if the Al^3+^ ions on the sapphire surface produced by the laser processing are coordinated or covered by other substances to block their chemical links with COO^−^ group and finally inhibit the subsequent adsorption of C-C/C-H, the observation of the extremely superhydrophilic can be possibly well maintained. Towards this goal, we chose the TiO_2_ material with the phototropic superhydrophilic property to hinder the adsorption of Al^3+^ and COO^−^, the preparation of which was described in Section 2.3. The annealing treatment not only transferred the TiO_2_ precursor into anatase crystals but also presented Al^3+^ doping into them. In other words, the naked Al^3+^ ions on the sapphire surfaces can be coordinated via such a process, which consequently hinders the chemisorption of COO^−^ for the transition of the superhydrophilic into the superhydrophobic. For convenience, here we would like to call the laser-processed surface with the TiO_2_ varnish as “Laser-TiO_2_” surface.

**Figure 3:**
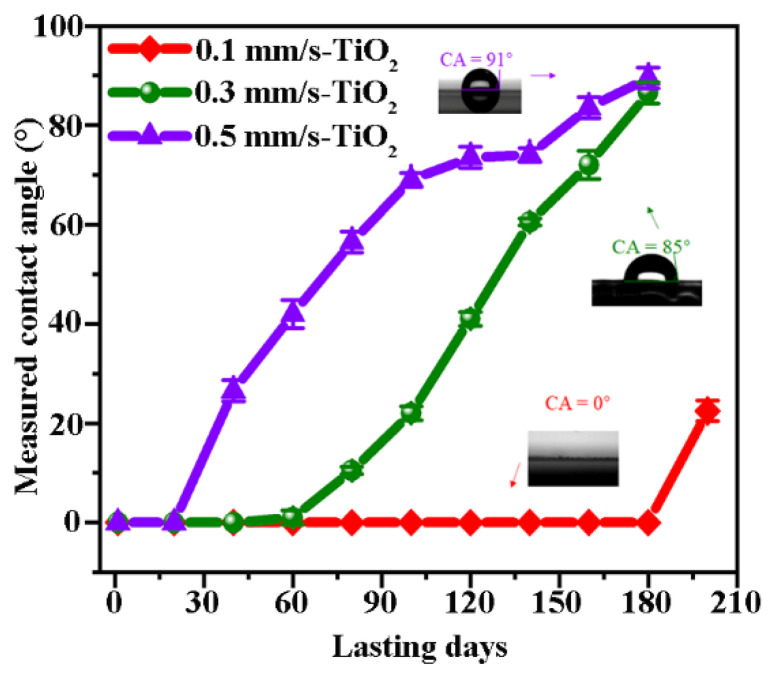
Measured evolution of the superhydrophilic persistence on the surface of Laser-TiO_2_ samples with time elapsing.

As a result, the superhydrophilicity for all the samples becomes evidently prolonged after the varnish of TiO_2_, and their durable performances depend on the morphology of the surface structures induced by different laser-scanning speeds. Figure 3 illustrates the measured evolution of the contact angle for the different samples with the storing days without the additional UV illumination. In comparison to the results without the TiO_2_ varnish (see Figure S2 from the Supplementary Materials), we find that at V = 0.5 mm/s the extreme superhydrophilicity of the Laser-TiO_2_ surfaces is prolonged to about half a month, while at V = 0.3 mm/s the persistent time of the extreme superhydrophilic can be extended to about 60 days. Remarkably, in the case of V = 0.1 mm/s, the superhydrophilic performance with CA = 0° can be well maintained as long as 180 days (see video S2 from the Supplementary Materials), and the superhydrophilic behavior completely disappears after 200 days (see Figure S4 from the Supplementary Materials). Furthermore, from the change of the measured curves at the larger scanning speeds, we also find that the transformation from the superhydrophilic to the hydrophobic effects is slowed down on the surface of the Laser-TiO_2_ samples.

### 3.3. Mechanisms of the Long-Term Persisting Superhydrophilic Effect

**Figure 4:**
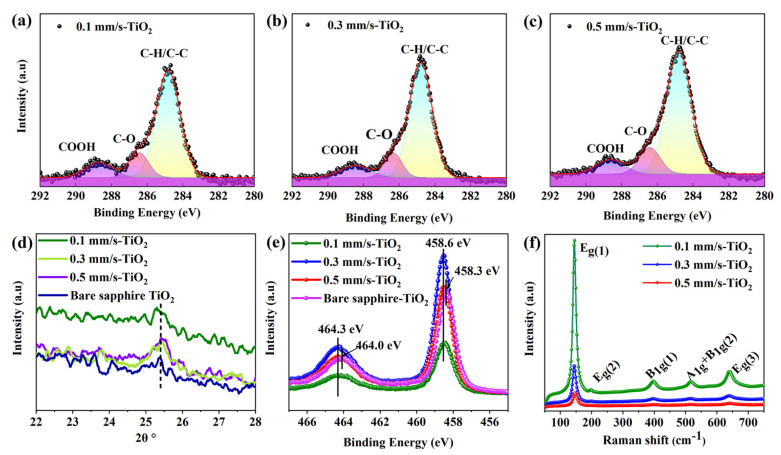
Characterization of the chemical change on Laser-TiO_2_ surfaces. (**a**–**c**) Measured XPS results of C 1 s spectra for the Laser-TiO_2_ surfaces with different scanning speeds; (**d**–**f**) Measured XRD, XPS (Ti 2 p), Raman spectra for the Laser-TiO_2_ surfaces with different scanning speeds.

To comprehensively understand the underlying mechanisms of this strategy, we performed XRD, XPS and Raman measurements of the Laser-TiO_2_ surfaces, as shown in Figure 4. From the XPS results of C 1 s in Figure 4a–c, we easily find that the adsorption of hydrophilic carboxyl -COOH (288.5 eV) happens on all the samples [47], which makes a sharp contrast to the observed absorption of the COO^−^ group without the varnish of TiO_2_ (see Figure S3a–c from the Supplementary Materials). Accordingly, the terminal parts of the long chain of amphiphilic hydrocarbon derivatives are no longer allowed to physically adsorb the non-polar saturated alkanes, leading to the unlikely transformation of the superhydrophilic into the hydrophobic on the surface. This indicates that the laser-induced Al^3+^ ions are not present separately anymore and they are probably coordinated by doping with TiO_2_ material, as shown in Figure 2b.

In addition, from the results of the XRD measurement in Figure 4d, we can see the characteristic peak at 2θ = 25.4° (corresponding to a <101> crystal plane of the anatase), which indicates the existence of the anatase TiO_2_ after the annealing process. Compared with both situations of V = 0.3 mm/s and 0.5 mm/s, a blue shift of the peak position happening for V = 0.1 mm/s suggests more Al^3+^ ions dopping into the titanium dioxide [48]. Furthermore, the measured XPS results of Ti 2 p in Figure 4e show that, for the bare sapphire surface, the peaks at 458.3 eV and 464 eV are attributed to the Ti-O-Ti bond in the anatase, whereas for the Laser-TiO_2_ samples, the two peak positions of Ti 2 p shift toward the higher binding energies of 458.6 eV and 464.3 eV, respectively, which also imply the doping of Al^3+^ ions into the TiO_2_ material [49]. In particular, the emergence of the binding energy at 458.6 eV can be attributed to the formation of the Al_2_TiO_5_ material [50], which provides more strong evidence for Al^3+^ ions doping into the TiO_2_ substance.

In terms of TiO_2_ doped by metal ions, many previous studies have confirmed that this would help to form the impurity energy levels within the bandgap and act as capture centers for the photogenerated electrons (or holes), so that the recombination of the photogenerated electron-hole pairs can be reduced to prolong the hydrophilicity of the material surface [51,52,53]. Remarkably, the decreasing in the bandgap can extend the photo response of TiO_2_ to the visible light region [54], thus improving the superhydrophilic effect without the UV light illumination.

Figure 4f shows the Raman spectra of the samples. The characteristic peaks at 144 cm^−1^, 196 cm^−1^, 638 cm^−1^ and 394 cm^−1^ belong to the active E_g(1)_, E_g(2)_, E_g(3)_ and B_1g_ vibrational modes of the anatase material, respectively, and the 516 cm^−1^ peak represents a dual vibrational A_1g_ and B_1g_ modes [55]. More interestingly, the available Raman peaks for Laser-TiO_2_ samples appear to be different in both the sharpness and the intensity with variable scanning speeds. For example, the peaks for V = 0.1 mm/s are much sharper and intense than those of the V = 0.3 mm/s and 0.5 mm/s cases. These results indicate that, for the surface with the small scanning speed, the growth of TiO_2_ substance tends to have the high crystallinity after the high-temperature annealing, which consequently slows down the electron-hole recombination rate to prolong the hydrophilicity.

**Figure 5:**
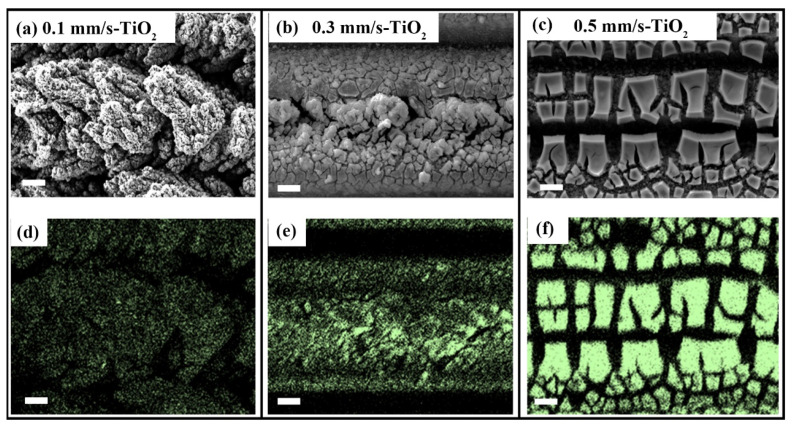
Characterization of the surface morphology and the elemental distribution for the Laser-TiO_2_ samples. (**a**–**c**) Variable morphology profiles at three different scanning speeds after femtosecond laser processing; (**d**–**f**) Observation of the elemental distribution patterns on the surfaces of three different scanning speeds. The green color for Ti element. Scale bars are 10 µm.

Apart from affecting the crystallinity of TiO_2_, the doping of metal ions also tends to inhibit the grain growth of TiO_2_ and prevent its transformation into rutile during the annealing process. Figure 5a–c show the surface morphology of the Laser-TiO_2_ sample. The surface with V = 0.1 mm/s is covered by many micron-scale clusters consisting of many nanoparticles to show a hierarchical structure, i.e., it has a larger roughness in surface morphology. This composite structure would like to exhibit the stronger capillary force. Through comparing their element distribution situations shown in Figure 5d–f, we find that, at the small laser-scanning speed of 0.1 mm/s, the ultrafine grains of TiO_2_ are almost uniformly dispersed among the multi-layer microstructures, almost without sacrifice of the surface roughness. However, for the larger scanning speeds, especially for the case of V = 0.5 mm/s, the evident aggregation of the TiO_2_ substance within the laser-ablated trenches would like to decrease the surface roughness or make the surface appearance smoother (compared with Figure S1 from the Supplementary Materials). Obviously, the achieved TiO_2_ crystals on the hierarchical structures can inevitably allow more absorption of photons.

**Figure 6:**
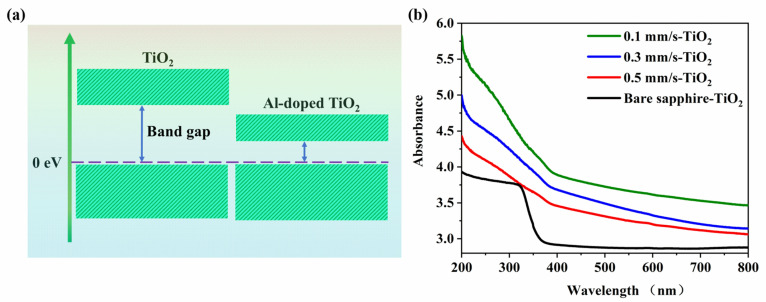
(**a**) Modified energy band structure of TiO_2_ by doping of Al^3+^ ions; (**b**) Measured absorption spectra for the surface of Laser-TiO_2_ samples.

According to the theoretical calculations on the energy band structure change of the anatase TiO_2_ after the metal ions doping [56], we can deduce that the bandgap of our Al^3+^ doped TiO_2_ film presents a decreasing trend, as shown in Figure 6a. In fact, such a modulation of the energy band was further confirmed by the comparative measurements of the UV-Vis absorption spectroscopy for the different samples, and the results are shown in Figure 6b. It is seen that for the surface of the TiO_2_- varnished bare sapphire material, the strong optical absorption occurs only in the UV range and presents a sharp cut-off wavelength at around λ = 387 nm, in accordance with the previous report [57]. However, for the Laser-TiO_2_ surfaces, the enhanced optical absorption takes place within the whole UV-Vis range. Specifically, for the case of V = 0.1 mm/s the optical absorption within the visible range can unprecedentedly improve to near 40%, which certainly helps to make the superhydrophilic of TiO_2_ more sensitive to both the wavelength and the intensity of light. At the same time, because of the generation of the Al_2_TiO_5_ material with a narrower bandgap on the surface, which corresponds to a longer absorption cut-off wavelength stretching to a visible range [58], the optical absorption spectra of the Laser-TiO_2_ surface are reasonably expanded.

### 3.4. Potential Applications of the Superhydrophilic Laser-TiO_2_ Surface

**Figure 7:**
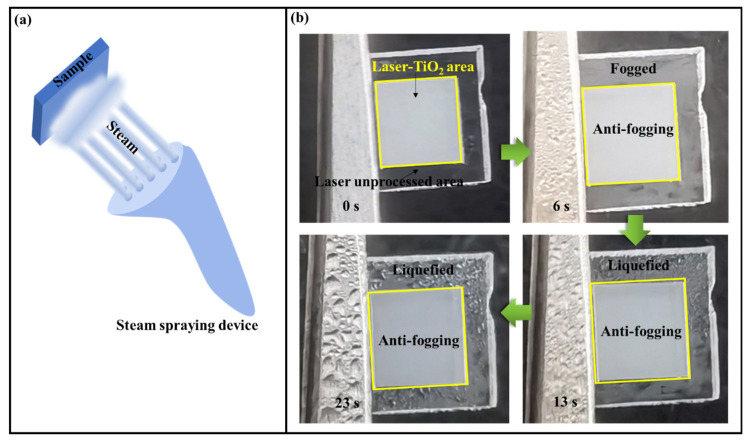
(**a**) A schematic illustration of the anti-fogging experiment; (**b**) Observation of anti-fogging behavior for the Laser-TiO_2_ surface through the steam spraying processes.

To further explore the potential applications of our samples, we carried out the anti-fogging tests with the continuous spraying of steam. The corresponding process and results are shown in Figure 7. At the spraying time of t = 6 s, the periphery surface of the laser processing begins to be fogged, while the centrally positioned Laser-TiO_2_ region is kept from the vapor condensation. As a matter of fact, this experimental observation can be more pronounced with the continuous increase in the spraying time up to t = 23 s, whereas the whole Laser-TiO_2_ surface can remain clear and dry without any water traces, in sharp contrast to the evident accumulation of the liquified water droplets on the periphery areas. Such phenomena suggest that the superhydrophilic Laser-TiO_2_ surface can quickly evaporate the water by its spreading into the large-area thin water film, leading to the so-called anti-fogging effect.

## 4. Conclusions

In summary, we comprehensively investigated how to make the widely used sapphire surface become the long-term sustainable superhydrophilic, especially with the contact angle of CA = 0°, by proposing a method of femtosecond laser processing associated with the subsequent varnish of TiO_2_. Our main results include two parts. (i) We explain why the superhydrophilic properties of the sapphire surface cannot usually persist in a long time only by the femtosecond laser processing method. This is because a large number of uncoordinated Al^3+^ ions can be generated through the femtosecond laser ablation, and subsequently their chemisorption with the COO^-^ group tend to develop a carboxylate layer, whose terminal is apt to adsorb more hydrophobic groups over time. Therefore, the initially observed superhydrophilic effect eventually transfers into the hydrophobic surface. (ii) In order to effectively extend the superhydrophilic persistence (especially with CA = 0°) of the sapphire surface after the laser processing, we employed spin-coating of TiO_2_ on the laser-structured surface and the subsequent heating treatment. This approach eventually resulted in the remarkable long-term superhydrophilic persistence of as long as 180 days. After comprehensive investigations, we believe that the coordination of Al^3+^ ions with TiO_2_ via doping tends to prevent the chemisorption of COO^−^ groups and the further adsorption of other hydrophobic groups. Meanwhile, the optical response of the varnished sample surface can be extended into the whole UV-Vis wavelength range. Moreover, the doping of metal ions also inhibited the growth of the anatase grains, which leaves the high degree of the surface roughness to inevitably increase the absorption of photons. As a result, the superhydrophilic property of Laser-TiO_2_ sample was achieved to persist for an unprecedentedly long time.

Finally, the potential applications of the samples were explored through the continuous spraying of steam on them. It was found that with the variation of spray time, the Laser-TiO_2_ surface showed an excellent anti-fogging performance due to its excellent superhydrophilic property. We hope all these investigations will benefit the design and fabrication of the superhydrophilic surfaces for future usages.

## Data Availability

Not applicable.

## Supplementary Materials

The following supporting information can be downloaded at: https://www.mdpi.com/article/10.3390/nano12193403/s1, Figure S1: Observation of the surface morphologies after femtosecond laser processing with different scanning speeds (a–c). Scale bars are 30 μm.; Figure S2: Measured superhydrophilic permanence over the time for three different samples without TiO2 varnish. Superhydrophilic (CA = 0°) property of V=0.1, 0.3, 0.5 mm/s can be maintained for 45 days, 17 days and a few days, respectively; Figure S3: Measured XPS results of C 1s spectra for the laser treated samples with different scanning speeds (a–c); Figure S4: Measured XPS results of the sample surfaces before and after losing the superhydrophilicity. Clearly, there is a significant increase in the hydrophobic group C-C/C-H; moreover, the atomic percentage of the carbon element on the surface is increased by 9.42%. This indicates that some uncoordinated Al^3+^ ions are still present on the structured surface; Video S1: A superhydrophilic surface with CA = 0°on the sapphire material by the laser ablation processing. Video S2: The superhydrophilic performance with CA = 0° can be well maintained as long as 180 days.
