# Supplementary material for: Long-Time Persisting Superhydrophilicity on Sapphire Surface via Femtosecond Laser Processing with the Varnish of TiO2
Source: Nanomaterials (Basel). 2022 Sep 28;12(19):3403. doi: 10.3390/nano12193403 (PMC9565462; doi:10.3390/nano12193403)
Supplement: Supplementary file 1 [file nanomaterials-12-03403-s001.zip › nanomaterials-1898819-supplementary/nanomaterials-1898819 SI conversion/nanomaterials-1898819 supplementary-done.pdf]

nanomaterials

Supplementary Materials

Long-Time Persisting Superhydrophilicity on Sapphire Surface via Femtosecond Laser Processing with the Varnish of TiO_2

Dandan Yan [1,2], Zhi Yu [1], Tingting Zou [1], Yucai Lin [1,2], Wenchi Kong [1,2] and Jianjun Yang [1,*]

1 GPL Photonics Laboratory, State Key Laboratory of Applied Optics, Changchun Institute of Optics, Fine Mechanics and Physics (CIOMP), Chinese Academy of Sciences (CAS), Changchun 130033, China; yandandan18@mails.ucas.ac.cn (D.Y.); zyu@imr.ac.cn (Z.Y.); zoutingting@ciomp.ac.cn (T.Z.); linyucai20@mails.ucas.ac.cn (Y.L.); kongwenchi17@mails.ucas.ac.cn (W.K.)
2 Center of Materials Science and Optoelectronics Engineering, University of Chinese Academy of Sciences, Beijing 100049 China.
* Correspondence: jjyang@ciomp.ac.cn

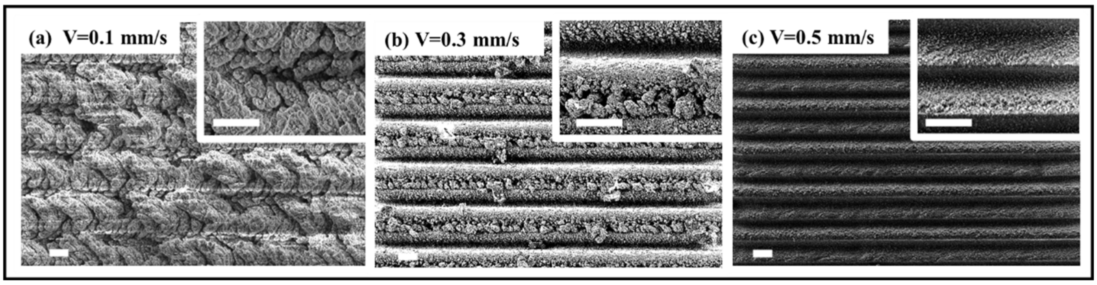

Figure S1. Observation of the surface morphologies after femtosecond laser processing with different scanning speeds (**a**–**c**). Scale bars are 30 µm.

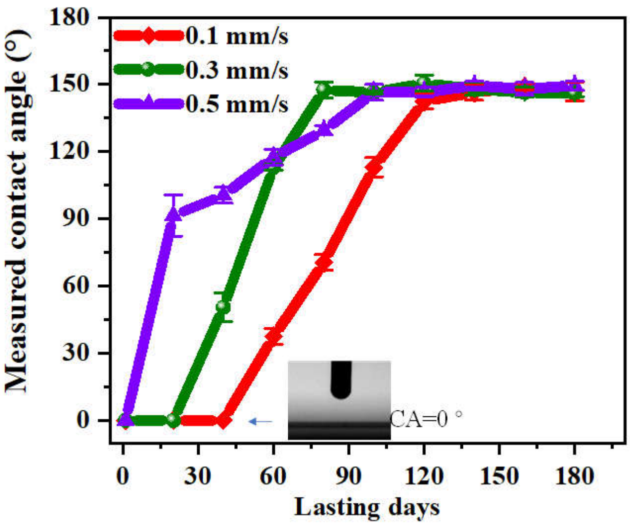

Figure S2. Measured superhydrophilic permanence over the time for three different samples without TiO_2 varnish. Superhydrophilic (CA = 0°) property of V=0.1, 0.3, 0.5 mm/s can be maintained for 45 days, 17 days and a few days, respectively.

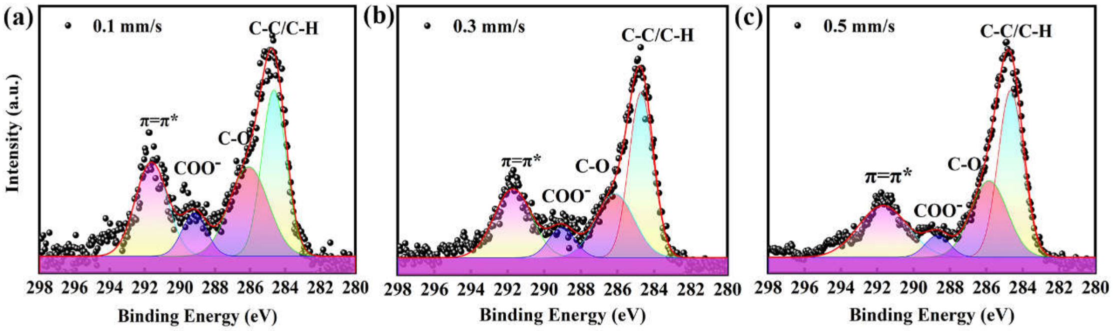

Figure S3. Measured XPS results of C 1s spectra for the laser treated samples with different scanning speeds (**a**–**c**).

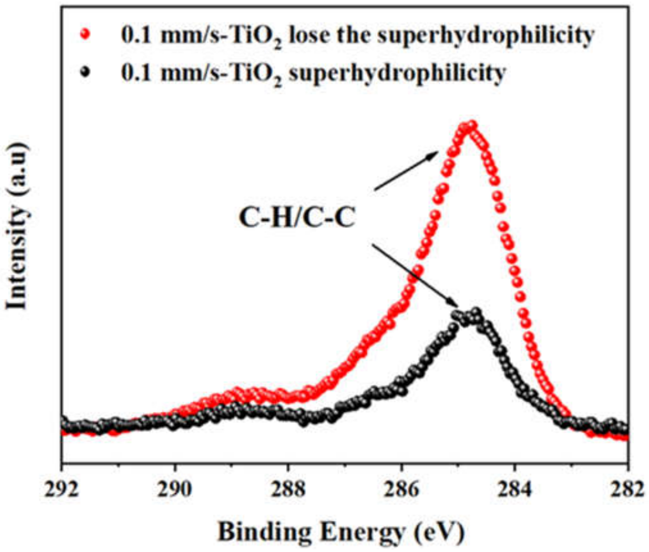

Figure S4. Measured XPS results of the sample surfaces before and after losing the superhydrophilicity. Clearly, there is a significant increase in the hydrophobic group C-C/C-H; moreover, the atomic percentage of the carbon element on the surface is increased by 9.42%. This indicates that some uncoordinated Al^{3+} ions are still present on the structured surface.
